# Bigenomic transcriptional regulation of all thirteen cytochrome c oxidase subunit genes by specificity protein 1

**DOI:** 10.1098/rsob.120176

**Published:** 2013-03

**Authors:** Shilpa S. Dhar, Kaid Johar, Margaret T. T. Wong-Riley

**Affiliations:** Department of Cell Biology, Neurobiology and Anatomy, Medical College of Wisconsin, 8701 Watertown Plank Road, Milwaukee, WI 53226, USA

**Keywords:** cytochrome c oxidase, depolarization, mitochondria, transcription factor, tetrodotoxin, specificity protein 1

## Abstract

Cytochrome c oxidase (COX) is one of only four known bigenomic proteins, with three mitochondria-encoded subunits and 10 nucleus-encoded ones derived from nine different chromosomes. The mechanism of regulating this multi-subunit, bigenomic enzyme is not fully understood. We hypothesize that specificity protein 1 (Sp1) functionally regulates the 10 nucleus-encoded *COX* subunit genes directly and the three mitochondrial *COX* subunit genes indirectly by regulating mitochondrial transcription factors A and B (*TFAM*, *TFB1M* and *TFB2M*) in neurons. By means of *in silico* analysis, electrophoretic mobility shift and supershift assays, chromatin immunoprecipitation, RNA interference and over-expression experiments, the present study documents that Sp1 is a critical regulator of all 13 *COX* subunit genes in neurons. This regulation is intimately associated with neuronal activity. Silencing of Sp1 prevented the upregulation of all *COX* subunits by KCl, and over-expressing Sp1 rescued all *COX* subunits from being downregulated by tetrodotoxin. Thus, Sp1 and our previously described nuclear respiratory factors 1 and 2 are the three key regulators of all 13 *COX* subunit genes in neurons. The binding sites for Sp1 on all 10 nucleus-encoded *COX* subunits, *TFAM*, *TFB1M* and *TFB2M* are highly conserved among mice, rats and humans.

## 2. Introduction

Neurons are highly dependent on ATP for their activity and functions [1,2]. Approximately 90 per cent of ATP generated in the brain is synthesized in the mitochondria via oxidative phosphorylation [3]. Eukaryotic cytochrome c oxidase (COX) is the terminal enzyme of the energy-transducing mitochondrial electron transport chain and is embedded in the inner mitochondrial membrane, where it mediates the transfer of electrons from reduced cytochrome c to molecular oxygen. COX also engages in proton pumping in the establishment of an electrochemical gradient for the synthesis of ATP. COX is a complex of 13 subunits; the largest three are encoded in the mitochondrial DNA and the remaining 10 are encoded in the nuclear genome [4]. To form a functional holoenzyme with 1 : 1 stoichiometry of all 13 subunits, exact coordination is essential between the two genomes. The mechanism of regulating this ancient, multi-subunit, bigenomic enzyme critical for cell survival is only beginning to be probed.

Two redox-responsive transcription factors, nuclear respiratory factors 1 and 2 (NRF-1 and NRF-2), have been found to be mediators of such bigenomic coordination in neurons. Specifically, they both regulate the expression of all 10 nucleus-encoded *COX* subunit genes [5–7], and indirectly regulate the three mitochondria-encoded *COX* subunit genes by activating mitochondrial transcription factors A and B (*TFAM*, *TFB1M* and *TFB2M*) [8,9]. Moreover, both NRF-1 and NRF-2 respond to and are regulated by neuronal activity, and changes in their mRNA and protein levels precede those of COX [6,10–12]. Besides NRF-1 and NRF-2, specificity protein 1 (Sp1) has been postulated to bind to the promoters of a few *COX* subunit genes [13–20] and *TFAM* [21,22]. However, none of these putative sites has been functionally characterized. The goal of the present study was to test our hypothesis that Sp1 is another bigenomic coordinator that regulates all 13 *COX* subunit genes.

By means of multiple approaches, *in silico* analysis, electrophoretic mobility shift and supershift assays, chromatin immunoprecipitation (ChIP), RNA interference and over-expression experiments, we document in this study that Sp1 functionally regulates all 13 *COX* subunit genes in neurons.

## 3. Results

### 3.1. *In silico* promoter analysis

Proximal promoters of murine nucleus-encoded *COX* subunits (*4i1*, *5a*, *5b*, *6a1*, *6b*, *6c*, *7a*, *7b*, *7c* and *8a*), *TFAM, TFB1M* and *TFB2M* genes with DNA sequence 1 kb 5′ upstream and 500 bps beyond 3′ of transcription start points (TSPs) were analysed *in silico* for potential Sp1-binding sites (table 1). Promoters for *COX4i1*, *5a*, *5b*, *6a1*, *6c*, *7a*, *7c* and *8a* showed a typical Sp1 sequence motif ‘GGGCGG’ or ‘CCCGCC’, whereas *COX6b* and *7b* had an atypical sequence of ‘GGGCGT’ or ‘GGGCGA’.

**Table 1.** EMSA probes. Positions of probes are given relative to TSP. Putative Sp1-binding sites are in boldface. Mutated nucleotide sequences are underlined.

gene	position	sequence
*COX4i1*	+23/+43	F: 5′-TTTTGGTGTAGA**GGGCGG**TCGCGGC-3′
		R: 5′-TTTTGCCGCGA**CCGCCC**TCTACACC-3′
*COX5a*	−20/+2	F: 5′-TTTTCTTATGCT**CCGCCC**AGCGTGCG-3′
		R: 5′-TTTTCGCACGCT**GGGCGG**AGCATAAG-3′
*COX5b*	+40/+59	F: 5′-TTTTGGAAGT**CCCGCCC**ATCTTGC-3′
		R: 5′-TTTTGCAAGATGGGCGGGACTTCC-3′
*COX6a1*	−53/−33	F: 5′-TTTTCTTTGT**CCCGCCC**CCTTCGCC-3′
		R: 5′-TTTTGGCGAAGGGGGCGGGACAAAG-3′
*COX6b*	−81/−61	F: 5′-TTTTGGAAGT**GGGCGT**GGCCAAAGC-3′
		R: 5′-TTTTGCTTTGGCCACGCCCACTTCC-3′
*COX6c*	+185/+204	F: 5′-TTTTTCGTGG**GGGCGG**CGAACTAG-3′
		R: 5′-TTTTCTAGTTCGCCGCCCCCACGA-3′
*COX7a2*	−47/−28	F: 5′-TTTTGGCA**GGGCGG**AGCCAAGTGG-3′
		R: 5′-TTTTCCACTTGGCTCCGCCCTGCC-3′
*COX7b*	+183/+202	F: 5′-TTTTATGT**GGGCGA**GTTCTTTACA-3′
		R: 5′-TTTTTGTAAAGAACTCGCCCACAT-3′
*COX7c*	−48/−29	F: 5′-TTTTCAGAG**CCCGCC**GGTTGAGTA-3′
		R: 5′-TTTTTACTCAACCGGCGGGCTCTG-3′
*COX8a*	−49/−30	F: 5′-TTTTGCCCA**GGCGGG**CGAGGGGGC-3′
		R: 5′-TTTTGCCCCCTCGCCCGCCTGGGC-3′
Sp-1 mutant (*COX4i1*)	+23/+43	F: 5′-TTTTGGTGTAGA**GGAAAA**TCGCGGC-3′
		R: 5′-TTTTGCCGCGA**TTTTCC**TCTACACC-3′
*GM3 synthase*	−58/−38	F: 5′-TTTTGCGCGAC**CCCGCCC**CCGCCTA-3′
		R: 5′-TTTTTAGGCGGGGGCGGGGTCGCGC-3′

### 3.2. *In vitro* binding of specificity protein 1 to promoters

**Figure 1.**
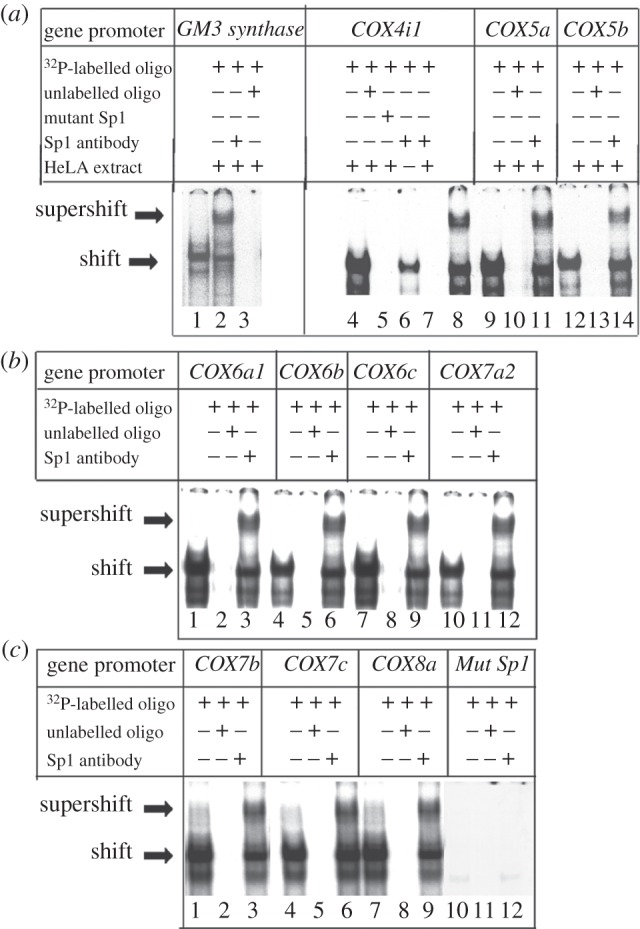
*In vitro* binding of Sp1 on *COX* subunit genes as measured with EMSA and supershift assays. ^32^P-labelled oligonucleotides, excess unlabelled oligos specific for each promoter as competitors, excess unlabelled mutant Sp1 as competitors, HeLa extract and Sp1 antibodies are indicated by a+or a−sign. Arrowheads indicate Sp1 shift and supershift complexes. The positive control, *GM3 synthase*, shows shift ((*a*), lane 1) and supershift ((*a*), lane 2) bands. When excess unlabelled competitor was added, it did not yield any band ((*a*), lane 3). All 10 *COX* subunit promoter sequences with putative Sp1-binding site showed specific shift and supershift bands that were eliminated by excess unlabelled competitors ((*a*), lanes 9–14; (*b*), all lanes and (*c*), lanes 1–9). Excess unlabelled but mutated Sp1 site could not compete ((*a*), lane 6). Labelled oligos with Sp1 antibodies alone with no HeLa extract did not yield any band ((*a*), lane 7). Labelled mutated Sp1 site on *COX4i1* was used as a negative control and it did not yield any band ((*c*), lanes 10–12).

*In vitro* electrophoretic mobility shift assays (EMSAs) were carried out using ^32^P-labelled probes (table 1) to determine the specificity of Sp1 binding to promoters of murine *COX* subunit genes (figure 1*a–c*). *GM3 synthase* promoter with a known Sp1-binding site at position −34/−55 served as a positive control [23] and it formed specific DNA/Sp1 shift and supershift complexes (figure 1*a*, lanes 1 and 2, respectively). When an excess of unlabelled probe was added as a competitor, no shift band was formed (figure 1*a*, lane 3). When labelled oligonucleotides were incubated with Sp1 antibody without HeLa nuclear extract, neither shift nor supershift band was observed (figure 1*a*, lane 7 for *Cox4i1*-specific oligo), ruling out non-specific antibody–oligo interactions. As shown in figure 1*a–c*, all 10 murine *COX* promoters formed specific DNA/protein shift complexes when incubated with purified HeLa nuclear extract (figure 1*a*, lanes 4, 9 and 12; figure 1*b*, lanes 1, 4, 7 and 10 and figure 1*c*, lanes 1, 4 and 7, respectively). These complexes were displaced by competition with excess unlabelled probes (figure 1*a*, lanes 5, 10 and 13; figure 1*b*, lanes 2, 5, 8 and 11 and figure 1*c*, lanes 2, 5 and 8, respectively), but was not displaced with unlabelled mutant *Sp1* probe (figure 1*a*, lane 6). Supershift assays using anti-Sp1 antibodies produced a supershift band of DNA/Sp1/antibody complex for each of the subunits (figure 1*a*, lanes 8, 11 and 14; figure 1*b*, lanes 3, 6, 9 and 12 and figure 1*c*, lanes 3, 6 and 9, respectively). Sp1 mutant showed neither shift nor supershift complexes, and unlabelled oligos also had no effect (figure 1*c*, lanes 10, 12 and 11, respectively).

### 3.3. *In vivo* occupancy of the promoters by specificity protein 1

ChIP assays were performed to verify possible Sp1 binding to all 10 *COX* promoters *in vivo. *β*-actin* exon 5 (*Actb*) served as a negative control, whereas *GM3* *synthase* with a known Sp1-binding site [23] served as a positive control. As a negative control, another immunoprecipitation from the same stock of cell lysate was done using anti-nerve growth factor receptor (NGFR) p75 antibodies. Polymerase chain reactions (PCRs) targeting regions of 10 *COX* subunit promoters surrounding putative Sp1-binding sites were carried out in parallel on chromatin immunoprecipitated from N2a cells. A 0.5 per cent dilution of input chromatin (i.e. *prior to* immunoprecipitation) was used as a standard to indicate the efficiency of the PCRs.

**Figure 2.**
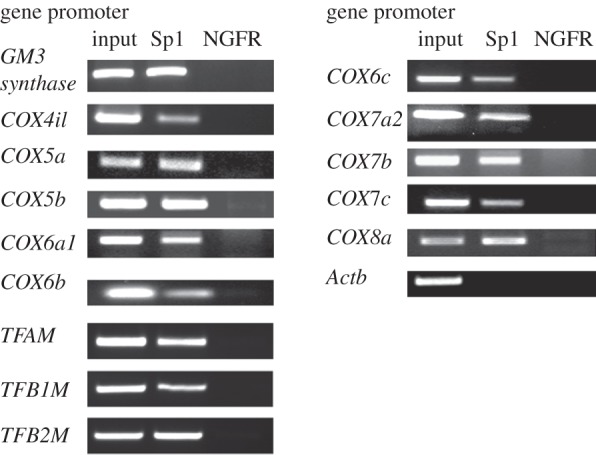
*In vivo* ChIP assays for Sp1 binding to nucleus-encoded *COX* subunit and mitochondrial transcription factor (*TFAM, TFB1M* and *TFB2M*) promoters. PCR reactions were performed on N2a cell chromatin precipitated with anti-Sp1 antibodies (Sp1 lanes) or anti-nerve growth factor receptor p75 antibodies (negative control, NGFR lanes). Control reactions were performed with input chromatin (input lanes). PCR products targeting *COX4i1*, *5a*, *5b*, *6a1*, *6b*, *6c*, *7a2*, *7b*, *7c* and *8a* promoters revealed that all 10 *COX* promoter DNAs co-immunoprecipitated with Sp1. Mitochondrial transcription factors (*TFAM, TFB1M* and *TFB2M*) also co-immunoprecipitated with Sp1 antibody. Reactions targeting *GM3 synthase* promoter was used as a positive control, and β-actin (*Actb*) was used as a negative control.

The proximal promoters of all 10 nucleus-encoded *COX* subunits *4i1*, *5a*, *5b*, *6a1*, *6b*, *6c*, *7a2*, *7b*, *7c* and *8a*, and mitochondrial transcription factors *TFAM*, *TFB1M* and *TFB2M* were co-immunoprecipitated with Sp1 antibodies and were amplified in semi-quantitative PCRs (figure 2). The amount of DNA precipitated by anti-Sp1 antibodies (Sp1 lanes) was greater than the amount precipitated by anti-NGFR (a negative control for background, NGFR lanes) for each of the 10 *COX* subunit promoters. *GM3 synthase* (positive control) showed a clear band, whereas *β-actin* (*Actb*, negative control) yielded no band with Sp1 (figure 2).

### 3.4. Effect of *Sp1* knock-down

**Figure 3.**
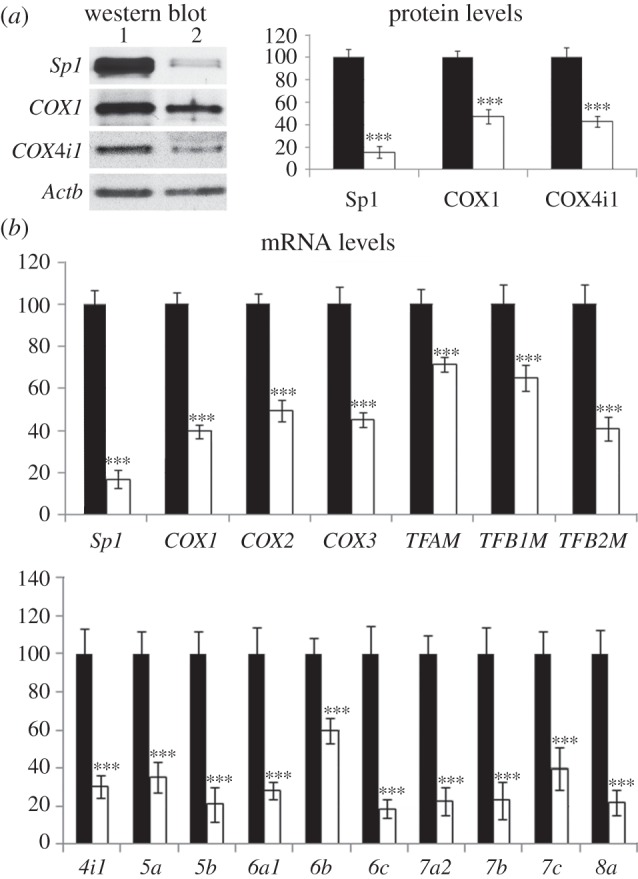
Knock-down of *Sp1* suppressed the expression of all 13 *COX* subunit genes and those of three mitochondrial transcription factors. N2a cells were transfected with shRNA against *Sp1* or with scrambled shRNA. (*a*) Western blot and protein levels of Sp1, COX1 and COX4i1. Lane 1, scrambled vector; lane 2, *Sp1* shRNA. *Sp1* shRNA significantly reduced the levels of Sp1, COX1 and COX4i1 proteins when compared with those with scrambled vectors. (*b*) mRNA levels of three mitochondrial *COX* subunits, three mitochondrial transcription factors and 10 nuclear *COX* subunits. *Sp1* shRNA significantly downregulated the transcripts for *Sp1*, three mitochondrial *COX* subunits, three mitochondrial transcription factors and 10 nuclear *COX* subunits when compared with scrambled vectors. *n* = 5 for each data point; ***p < 0.001 when compared with scrambled vectors. Black bar, scrambled vector; white bar, *Sp1* shRNA.

Transfection with vectors containing *Sp1* shRNA resulted in an 83 per cent decrease in the level of Sp1 mRNA (*p* < 0.001, figure 3*b*) and an 82 per cent decrease in Sp1 protein (*p* < 0.001, figure 3*a*). Silencing of *Sp1* led to a decrease in the mRNAs of the three mitochondrial *COX* subunits (*p* < 0.001 for all, figure 3*b*), three mitochondrial transcription factors (*p* < 0.001 for all, figure 3*b*) and 10 nucleus-encoded *COX* subunits (*p* < 0.001 for all, figure 3*b*) relative to scrambled vectors. Silencing of *Sp1* also led to a 55 per cent decrease in the protein level of Cox1 (*p* < 0.001, figure 3*a*), and a 40 per cent decrease in the level of Cox4i1 protein (*p* < 0.001, figure 3*a*).

### 3.5. Effect of *Sp1* over-expression

**Figure 4.**
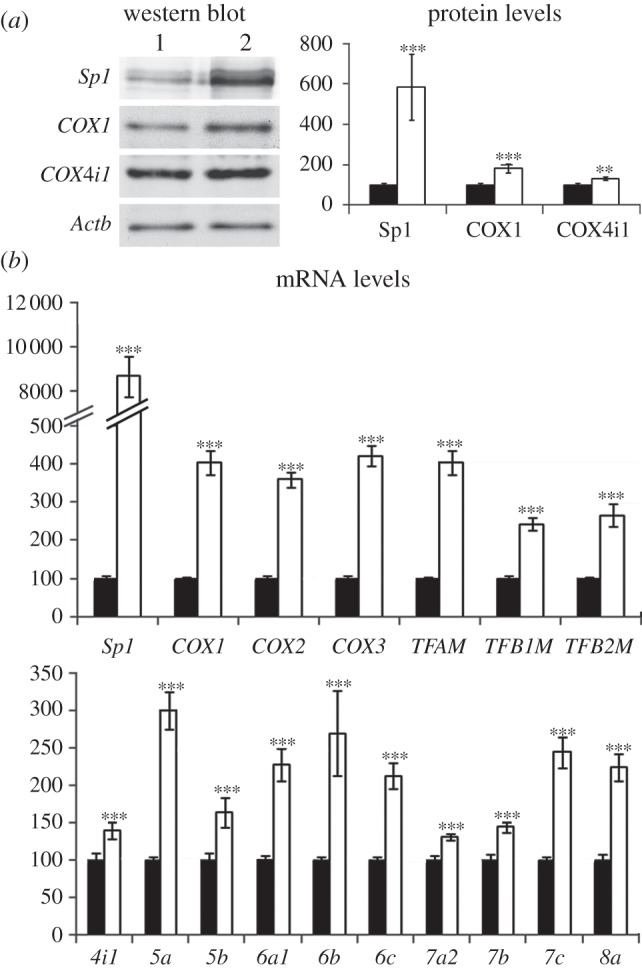
Over-expression of *Sp1* significantly increased the expression of all 13 *COX* subunit genes and those of three mitochondrial transcription factors. (*a*) Western blot and protein levels of Sp1, COX1 and COX4i1. Lane 1, empty vector; lane 2, *Sp1* over-expression. *Sp1* over-expression significantly increased the levels of Sp1, COX1 and COX4i1 proteins when compared with those with empty vectors. (*b*) mRNA levels of 13 *COX* subunits and mitochondrial transcription factors. *Sp1* over-expression significantly upregulated the mRNA levels of *Sp1*, three mitochondrial *COX* subunits, three mitochondrial transcription factors and 10 nuclear *COX* subunits when compared with empty vectors. *n* = 5 for each group. All *p*-values were compared with empty vectors. **p < 0.01 and ***p < 0.001 when compared with empty vectors. Black bar, empty vector; white bar, *Sp1* over-expression.

Transfection with vectors over-expressing *Sp1* led to an 80-fold increase in *Sp1* mRNA (*p* < 0.001, figure 4*b*) and a fivefold increase in Sp1 protein (*p* < 0.001, figure 4*a*). Over-expression of *Sp1* caused an increase in the mRNAs of three mitochondrial *COX* subunits (*p* < 0.001 for all, figure 4*b*), three mitochondrial transcription factors (*p* < 0.001 for all, figure 4*b*) and 10 nucleus-encoded *COX* subunits (*p* < 0.001 for all, figure 4*b*). Over-expression of *Sp1* also led to a 55 per cent increase in the protein level of Cox1 (*p* < 0.001, figure 4*a*) and a 40 per cent increase in the level of Cox4i1 protein (*p* < 0.01, figure 4*a*).

### 3.6. Effect of depolarization and impulse blockade

**Figure 5.**
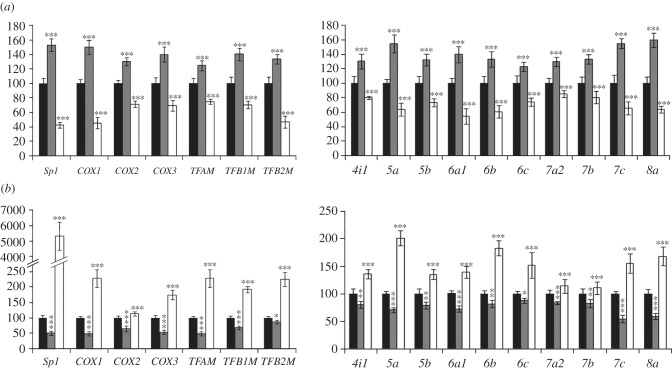
Effects of (*a*) KCl-induced depolarization and (*b*) TTX-induced impulse blockade on the expression of *COX* subunit mRNAs and those of mitochondrial transcription factors. (*a*) KCl-induced depolarization significantly increased the mRNA levels of *Sp1* itself, the three mitochondrial *COX* subunits, three mitochondrial transcription factors and 10 nuclear *COX* subunits. Neurons that were transfected with *Sp1* shRNA, on the other hand, were not able to upregulate any of these transcripts in the presence of KCl. Black bar, control; grey bar, control+KCl; white bar, *Sp1* shRNA+KCl. (*b*) TTX-induced impulse blockade led to a significant reduction of transcripts for *Sp1*, three mitochondrial *COX* subunits, three mitochondrial transcription factors and 10 nuclear *COX* subunits. However, the over-expression of *Sp1* rescued all of these transcripts from TTX-induced suppression. Black bar, empty vector; grey bar, empty vector+TTX; white bar, *Sp1* over-expression+TTX. *n* = 5 for each data point; *p < 0.05; **p < 0.01; ***p < 0.001 when compared with either scrambled or empty vector.

To determine if the expression of *COX* subunit genes was affected by depolarizing stimulation, cells were subjected to 20 mM of KCl for 5 h after incorporating *Sp1* shRNA. The KCl regimen was found previously to activate *COX* gene expression in primary neurons [10,11]. As shown in figure 5, depolarizing stimulation resulted in a significant increase in the mRNA levels of *Sp1* (*p* < 0.001, figure 5*a*), three mitochondrial *COX* subunits (*p* < 0.001 for all, figure 5*a*), three mitochondrial transcription factors (*p* < 0.001 for all, figure 5*a*) and 10 nucleus-encoded *COX* subunits (*p* < 0.001 for all, figure 5*a*). With *Sp1* silencing, both mitochondria-encoded and nucleus-encoded COX subunits as well as the three mitochondrial transcription factors failed to respond to KCl.

Tetrodotoxin (TTX) at 0.4 μM has been shown to decrease the levels of *COX* subunit mRNAs as well as COX enzyme activity *in vivo* and in primary neurons [24–26]. To evaluate the effect of impulse blockade on *COX* subunits, N2a cells were treated with TTX for 3 days. TTX reduced the mRNA levels of *Sp1* (*p* < 0.001, figure 5*b*), three mitochondrial *COX* subunits (*p* < 0.001 for all, figure 5*b*), three mitochondrial transcription factors (*p* < 0.001 for *TFAM* and *TFB1M*; *p* < 0.05 for *TFB2M,* figure 5*b*) and 10 nucleus-encoded *COX* subunits (*p* < 0.001 for *COX5a*, *6a1*, *7a2*, *7c* and *8a*; *p* < 0.01 for *COX4i1*, *5b, 6b* and *7b*; *p* < 0.05 for *COX6c*; figure 5*b*). This indicates an overall suppressive effect of TTX on all *COX* subunit gene expression in neurons. On the other hand, reductions in the mRNA levels of *Sp1* (*p* < 0.001, figure 5*b*), three mitochondrial *COX* subunits (*p* < 0.001 for all), three mitochondrial transcription factors (*p* < 0.001 for all, figure 5*b*) and 10 nucleus-encoded *COX* subunits (*p* < 0.001 for all, figure 5*b*) were rescued in cells that over-expressed *Sp1* (figure 5*b*).

### 3.7. Homology of specificity protein 1-binding sites

**Figure 6.**
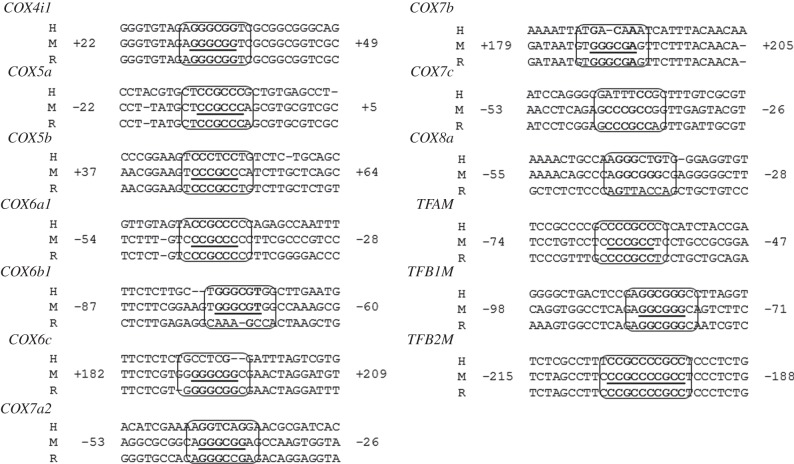
Aligned partial sequences of promoters for nucleus-encoded *COX* subunits and mitochondrial transcription factors (*TFAM*, *TFB1M* and *TFB2M*) from mice (M), rats (R) and humans (H). Conserved putative Sp1-binding sites are shown in solid boxes. Sp1-binding sites in mice are shown in bold and underlined.

The functional Sp1-binding sites on 10 nucleus-encoded *COX* subunits, *TFAM*, *TFB1M* and *TFB2M* are conserved among humans, mice and rats (figure 6). Sp1 sites had a relatively high degree of homology (60–100%; mostly 80–90%) among the three species.

## 4. Discussion and conclusions

Using multiple molecular and biochemical approaches, including *in silico* analysis, electrophoretic mobility shift and supershift assays, ChIP assays, RNA interference and over-expression assays, we documented that Sp1 transcription factor regulates all 10 nucleus-encoded *COX* subunit genes directly and three mitochondrial-encoded *COX* genes indirectly by regulating the three transcription factors important in the transcription of mitochondrial DNA. Knocking down Sp1 resulted in a significant reduction in the expression of all 10 nucleus-encoded and three mitochondria-encoded *COX* subunit genes, and *TFAM*, *TFB1M* and *TFB2M* genes in neurons. Over-expression of *Sp1* resulted in a significant upregulation of *Sp1* itself, all 10 nucleus-encoded and three mitochondria-encoded *COX* subunit genes, and *TFAM*, *TFB1M* and *TFB2M* genes in neurons. The expression of all *COX* subunits and their transcription factor, *Sp1*, are tightly coupled to neuronal activity. The Sp1-binding sites on promoters of nuclear *COX* subunit, *TFAM, TFB1M* and *TFB2M* genes are conserved among mice, rats and humans. It is noteworthy that *COX6b* and *7b* had an atypical Sp1-binding motif (‘GGGCGT’ and ‘GGGCGA’, respectively), whereas other *COX* promoters as well as those for *TFAM*, *TFB1M* and *TFB2M* all had a typical Sp1-binding sequence.

Sp1 is the first C_2_H_2_-type zinc finger transcription factor to be isolated and cloned from mammalian cells. Sp1 recognizes CG-rich sequences in CpG islands and was initially recognized as a constitutive transcription activator of housekeeping genes and other TATA-less genes. Sp1 is ubiquitously expressed, and at least 12 000 Sp1 typical binding sites have been found in the human genome, most of which have been associated with genes involved in many cellular functions [27–30]. Knock-out of Sp1 is embryonically lethal [27,28]. Earlier studies have shown the presence of Sp1-binding sites on the promoter regions of some *COX* subunit genes. These include *COX4i1* in humans, mice and rats [14,31,32], *COX5b* in mice and humans [16,18,20] and *COX6a1* in rats [13]. Putative Sp1-binding sites have also been reported on nucleus-encoded *TFAM* gene [21,22]. However, functional characterization has not been done on any of these sites. The present study documented that Sp1 bigenomically regulates all 13 *COX* subunit genes, making it an important regulator of genes involved in the production of cellular energy. The expression of *Sp1* itself is tightly regulated by neuronal activity. Likewise, the regulation of all *COX* subunit genes and mitochondrial transcription factors is strongly dictated by neuronal activity, consistent with our earlier studies [10,11,24,26]. Depolarizing stimulation failed to increase their expression in the presence of *Sp1* siRNA, and *Sp1* over-expression rescued their downregulation induced by impulse blockade. These findings substantiate the important role of *Sp1* in regulating *COX* subunit genes in an activity-dependent manner.

COX is a critical terminal enzyme of the mitochondrial electron transport chain, without which oxidative metabolism cannot be carried to completion and ATP cannot be generated [1,2,4]. The three mitochondria-encoded subunits of COX (COX1, COX2 and COX3) form the catalytic core of the enzyme, whereas the 10 nucleus-encoded subunits reportedly serve modulatory functions [33,34]. COXIV binds ATP directly and regulates COX activity based on the intra-mitochondrial ATP/ADP ratio [35,36]. COX5b and COX6a are also involved in the regulation of COX activity [37,38]. COX5a has a binding site for 3,5-diiodothyronine (T2) and relieves the inhibition of COX enzyme induced by intra-mitochondrial ATP [39]. COX6b is involved in the stabilization of the dimeric COX enzyme, and Cox6c provides a low-affinity binding site for cytochrome c [40]. Three of the nuclear subunit proteins (6a, 7a and 8) have tissue-specific isoforms and presumably regulate COX activity in different tissues based on tissue-specific requirements [33,41]. The functions of subunits Cox7a, 7b, 7c and 8a are not known with certainty.

The mechanism of regulating this multi-subunit, bigenomic and multi-chromosomal protein poses a major challenge. Recently, we demonstrated that all 10 nucleus-encoded subunits of *COX* are regulated by the transcription factors NRF-1 and NRF-2 [5–7]. Both factors also regulate indirectly the three mitochondria-encoded *COX* subunit genes by activating *TFAM, TFB1M* and *TFB2M* important for mitochondrial DNA transcription and replication [8,9]. The present study revealed that Sp1 is the third transcription factor known thus far to functionally regulate all 10 nucleus-encoded and three mitochondrial-encoded *COX* subunit genes in neurons *in vitro* and *in vivo*. Moreover, our recent study showed that all of the nucleus-encoded *COX* subunit transcripts and those for *TFAM*, *TFB1M* and *TFB2M* are all transcribed in the same transcription factory [42].

The coordinated regulation of all 13 *COX* subunit genes is likely to be complex and multifactorial, requiring various transcription factors and coactivators. The mechanism of coordinating multiple transcription factors can be complementary, concurrent and parallel or a combination of complementary and concurrent/parallel. In the case of NRF-1 and NRF-2, our recent study has shown that both NRF-1 and NRF-2 regulate the same set of genes in a parallel and concurrent manner [43]. Moreover, double knock-down of NRF-1 and Sp1 or of NRF-2 and Sp1 in primary neurons affected cell survival (Dhar and Wong-Riley 2012, unpublished data), indicating important roles of these factors in activating key metabolic genes that regulate cell growth, functioning and survival. Sp1-binding sites are in close proximity to those of NRF-1 and NRF-2 (Dhar and Wong-Riley 2012, unpublished data). Thus, Sp1 may physically interact with NRF-1 and/or NRF-2 in regulating some or all of the *COX* subunit genes. Future studies will be directed at evaluating such possible protein–protein interactions and possible colocalization of these factors in the same or different transcription factories. In addition to transcription factors, a transcriptional coactivator, peroxisome proliferator-activated receptor gamma coactivator 1α (PGC-1α), is also likely to be involved in the regulation of *COX* in neurons. PGC-1α coactivates NRF-1 and NRF-2 in the control of mitochondrial biogenesis [44,45] and its response to changes in neuronal activity precedes those of NRF-1 and NRF-2 [10,46].

In conclusion, our multi-faceted investigation revealed that Sp1, in addition to NRF-1 and NRF-2, is a critical regulator of all 13 *COX* subunit genes in neurons ([5–7], the present study).

## 5. Experimental procedures

### 5.1. *In silico* analysis of promoters of murine *COX* subunit genes

DNA sequences surrounding the TSPs of all 10 nucleus-encoded *COX* subunit genes, and *TFAM*, *TFB1M* and *TFB2M* were derived from the mouse, rat and human genome database in GenBank. These promoter sequences encompassed 1 kb upstream and up to 500 bps downstream (excluding protein-coding sequence) of the TSP of each subunit gene analysed. These sequences were aligned using Megalign, DNAStar Lasergene software. Putative Sp1-binding sequences ‘GGGCGG’ or ‘CCCGCC’ were searched using DNAStar Lasergene software. Regions of high homology were selected for experimental analyses.

### 5.2. Cell culture

Murine neuroblastoma (N2a) cells (ATCC, Manassas, VA, USA) were grown in Dulbecco's modified Eagle's medium supplemented with 50 units ml^−1^ penicillin, 100 μg ml^−1^ streptomycin (Invitrogen, Carlsbad, CA, USA) and 10 per cent foetal bovine serum (Sigma, St Louis, MO, USA) at 37°C in a humidified atmosphere with 5 per cent CO_2_.

### 5.3. Electrophoretic mobility shift and supershift assays

EMSAs to assay possible binding of Sp1 on all 10 *COX* subunit promoters were carried out with methods as described previously [7]. Briefly, oligonucleotide probes with putative Sp1-binding site on each promoter (table 1, based on *in silico* analysis) were synthesized, annealed and labelled by a Klenow fragment (Invitrogen) fill-in reaction with [α-^32^P] dATP (50 μCi/200 ng; Perkin-Elmer, Shelton, CT, USA). Each labelled probe was incubated with 2 µg of calf thymus DNA and 5 µg of HeLa nuclear extract (Promega, Madison, WI, USA) and processed for EMSA. Supershift assays were also performed, and in each reaction, 1–1.5 µg of Sp1-specific antibodies (polyclonal goat antibodies, Santa Cruz Biotechnology, Santa Cruz, CA, USA) were added to the probe/nuclear extract mixture and incubated for 20 min at room temperature. For competition, 100-fold excess of unlabelled oligonucleotides were incubated with nuclear extract before adding labelled oligonucleotides. Shift reactions were loaded onto 4 per cent polyacrylamide gel and run at 200 V for 2.5 h in 0.25× Tris/borate/EDTA (TBE) buffer. Results were visualized by autoradiography. Mouse *GM3 synthase* with Sp1-binding site at position −34/−55 was designed as previously described [23] and used as a positive control. Sp1 mutants with mutated sequences as shown in table 1 were used as negative controls.

### 5.4. Chromatin immunoprecipitation assays

ChIP assays were performed similar to those described previously [7]. Briefly, approximately 750 000 N2a cells were used for each immunoprecipitation and were fixed with 1 per cent formaldehyde for 10 min at room temperature. ChIP assay kits (Upstate, Charlottesville, VA, USA) were used with minor modifications. Following formaldehyde fixation, cells were resuspended in a swelling buffer (5 mM PIPES, pH 8.0, 85 mM KCl, 1% NP-40 and protease inhibitors added right before use) and homogenized 10 times in a small pestle Dounce tissue homogenizer (7 ml). Nuclei were then isolated by centrifugation before being subjected to sonication. The sonicated lysate was immunoprecipitated with either 0.2 µg of Sp1 polyclonal rabbit antibodies (H-225, sc-14027; Santa Cruz Biotechnology) or 2 µg of NGFR p75 polyclonal goat antibodies (C20, sc-6188; Santa Cruz Biotechnology). Semi-quantitative PCR was performed using 1/20th of precipitated chromatin. Primers targeting promoter sequences near TSP of *COX* subunit genes were designed (table 2). Mouse *GM3 synthase* promoter previously found to have Sp1 binding [23] was used as a positive control, and exon 5 of *β-actin* gene was used as a negative control (table 2). PCR reactions were carried out with the EX Taq hot-start polymerase (Takara Mirus Bio, Madison, WI, USA) with the following cycling parameters: 30 s denaturation at 94°C, 30 s annealing at 59.5°C and 20 s extension at 72°C (32–36 cycles per reaction). All reactions were hot-started by heating to 94°C for 120 s. Use of hot-start polymerase and PCR additives significantly improved the quality and reproducibility of ChIP. PCR products were visualized on 2 per cent agarose gels stained with ethidium bromide.

**Table 2.** Primers for ChIP assays. Positions of amplicons are given relative to TSP.

gene	sequence	amplicon length (bp)
*COX4i1*	F: 5′-GAAAACGTCTGCCGGAAAG-3′	284
	R: 5′-GTCACCTGCCACCGCTG-3′	
*COX5a*	F: 5′-CCACGCAGGAATGTTCACTA-3′	237
	R: 5′-ACGAGAAGCCGGTGTGAG-3′	
*COX5b*	F: 5′-GCGTTGTTAGACTCCCACCA-3′	200
	R: 5′-AGCTGGTCACGTACCTCCAG-3′	
*COX6a1*	F: 5′-GCTGACAAGCAGGGAGATG-3′	215
	R: 5′-GACGCCATCATGGAACTACA-3′	
*COX6b*	F: 5′-GCCCAGCAACAATAATAAGCA-3′	196
	R: 5′-TAGCAAAGACGCCAATGTCA-3′	
*COX6c*	F: 5′-GGTGAGAATGGTGGAGAGAGA-3′	299
	R: 5′-GCAAAATACAAGGGGAAACG-3′	
*COX7a2*	F: 5′-CGTTTGCTTTCCATTGTGATT-3′	219
	R: 5′-AAACGGAACTCCCTCCTAGC-3′	
*COX7b*	F: 5′-GATGTTGCCCTTAGCCAAAA-3′	194
	R: 5′-CTAGCTTCCCTTCCCAGTGA-3′	
*COX7c*	F: 5′-ACATTTCCCACAATCCATCG-3′	189
	R: 5′-ATGGCCGTACCACCTAACTC-3′	
*COX8a*	F: 5′-TGGGAGCAAAGGTGTCTCAT-3′	213
	R: 5′-GTCCAAGGTCAGGGAGTCAA-3′	
*TFAM*	F: 5′- GTGACACAAGCCGCAGCAC-3′	331
	R: 5′- CACTACCAGCGTGGGAACTCC-3′	
*TFB1M*	F: 5′- CTGAAAGAAGTAATGGACGCG-3′	340
	R: 5′- GTGGGACCTTGGAGAAGAC-3′	
*TFB2M*	F: 5′- GAGGATCGGACACCTCTAGC-3′	290
	R: 5′- CACATTTCACCACCACACTAGG-3′	
*GM3 synthase*	F: 5′-CACCTACTTCTCGGCTGGAG-3′	198
	R: 5′-AATTCAGCCCCGGACAGT-3′	
*β-actin* exon 5	F: 5′-GCTCTTTTCCAGCCTTCCTT-3′	187
	R: 5′-CGGATGTCAACGTCACACTT-3′	

### 5.5. Knock-down of *Sp1* with shRNA and KCl treatment

*Sp1* shRNA plasmids for mouse were obtained from Santa Cruz Biotechnology (sc-29488-SH) as a pool of three target-specific lentiviral vectors with H1 promoter and puromycin resistance. Each vector encompassed 19–25 nt (plus hairpin) shRNAs designed to knock-down gene expression of *Sp1*. Control shRNA plasmid-A (Santa Cruz Biotechnology; sc-108062) or scrambled shRNA served as negative controls. N2a cells were plated in 60-mm dishes at a density of 5 to 8 × 10^5^ cells/dish. They were cotransfected 3 days post-plating with *Sp1* shRNA at 1 µg per dish via Lipofectamine 2000. Empty vectors or scrambled shRNA vectors alone were used at the same concentrations as vectors with shRNA against *Sp1*. Puromycin at a final concentration of 0.5 µg ml^−1^ was added to the culture medium on the second day after transfection to select for purely transfected cells. Green fluorescence was observed to monitor transfection efficiency. Transfection efficiency for N2a cells ranged from 40 to 75 per cent. Transfected cells were selected by the addition of puromycin. This antibiotic selection yielded essentially 100 per cent transfected cells. After 48 h of transfection, cells were stimulated with 20 mM KCl in the culture media for 5 h as previously described [10,11]. Cells were harvested and processed for the isolation of RNAs and proteins.

### 5.6. *Sp1* over-expression and tetrodotoxin treatment

The human *Sp1* cDNA clones were obtained from Open Biosystems (Lafayette, CO, USA). The *Sp1* cDNA was cloned into pcDNA Dest40 vector using Gateway Multisite Cloning kit (Invitrogen) and according to the manufacturer's instructions. N2a cells were plated in six-well plates at a density of 5 to 8 × 10^6^ cells per well. They were transfected 1 day post-plating with 2 μg *Sp1*-containing plasmids using JetPrime reagent (Polyplus Transfection, New York, NY, USA). Empty vectors were used as a control and at the same concentration as vectors with Sp1 insert. Geneticin 500 μg ml^−1^ (Invitrogen) was added to the culture medium on the second day after transfection for the selection of transfected cells. Transfection efficiency for N2a cells ranged from 65 to 75 per cent. Transfected cells were selected using 250 μg ml^−1^ Geneticin. This antibiotic selection yielded essentially 100 per cent transfected cells. N2a cells transfected with *Sp1* were treated with 0.4 μM TTX in the culture media for 3 days. Cells were harvested and processed for the isolation of RNAs and proteins.

### 5.7. RNA isolation and cDNA synthesis

Total RNA was isolated by RNeasy kits (Qiagen, Valencia, CA, USA) according to the manufacturer's instructions. Three micrograms of total RNA were treated with DNase I and purified by phenol–chloroform. cDNA was synthesized using the iScript cDNA synthesis kit (Bio-Rad, Hercules, CA, USA) according to the manufacturer's instructions.

### 5.8. Real-time quantitative polymerase chain reaction

Real-time quantitative PCRs were carried out in a Cepheid Smart Cycler Detection system (Cepheid, Sunnyvale, CA, USA). SyBr Green (BioWhittaker Molecular Application, Rockland, ME, USA) and EX Taq real-time quantitative PCR hot-start polymerase were used following the manufacturer's protocols and as described previously [7]. Primer sequences are shown in table 3. PCR runs: hot start 2 min at 95°C, denaturation 10 s at 95°C, annealing 15 s according to the Tm of each primer and extension 10 s at 72°C for 15–30 cycles. Melt curve analyses verified the formation of single desired PCR product. Rat 18S was used as an internal control, and the 2^−*ΔΔ*CT^ method [47] was carried out for the relative amount of transcripts.

**Table 3.** Primers for real-time quantitative PCR. Positions of amplicons are given relative to TSP.

gene	sequence	amplicon (bp)	Tm (°C)
*COX1*	F: 5′-GCCTTTGCTTCAAAACGAGA-3′	105	58
	R: 5′-GGTTGGTTCCTCGAATGTGT-3′		
*COX2*	F: 5′-TCTCCCCTCTCTACGCATTC-3′	161	59
	R: 5′-CAGGTTTTAGGTCGTTTGTTG-3′		
*COX3*	F: 5′-ACTTCACCATCCTCCAAGC-3′	151	59
	R: 5′-TGTCGTAGTAGGCAAACAATAAGG-3′		
*COX4i1*	F: 5′-TCTACTTCGGTGTGCCTTCG-3′	253	59.5
	R: 5′-ACTCATTGGTGCCCTTGTTC-3′		
*COX5a*	F: 5′-GGAGTTGCGTAAAGGGATGA-3′	247	60
	R: 5′-CACTTTGTCAAGGCCCAGTT-3′		
*COX5b*	F: 5′-GGAGGTGGTGTCCCTACTGA-3′	241	59.5
	R: 5′-CAGCCAGAACCAGATGACAG-3′		
*COX6a1*	F: 5′-TCAACGTGTTCCTCAAGTCGC-3′	115	60
	R: 5′-AGGGTATGGTTACCGTCTCCC-3′		
*COX6b*	F: 5′-ATGGCCGAAGACATCAAGAC-3′	250	60
	R: 5′-CAGGAAATGTGCCTTCTGCT-3′		
*COX6c*	F: 5′-AGCGTCTGCGGGTTCATA-3′	154	60
	R: 5′-GCCTGCCTCATCTCTTCAAA-3′		
*COX7a2*	F: 5′-GAGGACCATCAGCACCACTT-3′	234	59.5
	R: 5′-TGGAGACTGGGATGACGAC-3′		
*COX7b*	F: 5′-CGAAGCATTCAGCAAGTGGT-3′	209	59.5
	R: 5′-TGGCATGACTACTGATCTCTCC-3′		
*COX7c*	F: 5′-TCTGCCTTCCGTCTCTGC-3′	145	60
	R: 5′-AGAAAGGAGCAGCAAATCCA-3′		
*COX8a*	F: 5′-TCCTGCTTCGTGTGTTGTCT-3′	70	59
	R: 5′-TCCCGCTTCTTGTAGCTTTC-3′		
*TFAM*	F: 5′-CAGGGCTGCAATTTTCCTAA-3′	141	58
	R: 5′-CCGAAGTGTTTTTCCAGCAT-3′		
*TFB1M*	F: 5′-AAGATGGCCCTTTCGTTTATGG-3′	102	59
	R: 5′-GACTGTGCTGTTTGCTTCCTG-3′		
*TFB2M*	F: 5′-CCAAAACCCATCCCGTCAAAT-3′	135	59
	R: 5′-AAGGGCTCCAAATGTGGAATAAA-3′		
*Actb*	F: 5′-GGCTGTATTCCCTCCATCG-3′	154	59.5
	R: 5′-CCAGTTGGTAACAATGCCATGT-3′		

### 5.9. Western blot

Proteins from *Sp1* shRNA and over-expression samples along with appropriate controls were loaded onto 10 per cent SDS-PAGE gel and electrophoretically transferred onto polyvinylidene difluoride membranes (Bio-Rad). Subsequent to blocking, blots were incubated in primary antibodies against Sp1 (1 : 1000; Santa Cruz), COX1 (1 : 1000; Molecular Probes, Life Technologies) and polyclonal antibodies that recognize mainly COX4i1 (1 : 1000) [48]. β-Actin (1 : 3000; Sigma) served as a loading control. Secondary antibodies used were goat anti-rabbit and goat anti-mouse antibodies (Vector Laboratories, Burlingame, CA, USA). Blots were then reacted with the enhanced chemiluminescent (ECL) reagent (Pierce, Rockford, IL, USA) and exposed to autoradiographic film (RPI, Mount Prospect, IL, USA). Quantitative analyses of relative changes were done with an Alpha Imager (Alpha Innotech, San Leandro, CA, USA).

### 5.10. Statistical analysis

Significance among group means was determined by analysis of variance (ANOVA). Significance between two groups was analysed by Student's *t*-test. *p*-values of 0.05 or less were considered significant.

## 6. Acknowledgements

Supported by National Institutes of Health grant no. R01 EY018441.
